# Supplementary material for: Successional Shifts in Soil C:N:P stoichiometry and its environmental controls during forest restoration in Huize County, Qujing, Yunnan Province
Source: PLoS One. 2026 Aug 21;21(8):e0356261. doi: 10.1371/journal.pone.0356261 (PMC13496410; doi:10.1371/journal.pone.0356261)
Supplement: Supplementary file 2 [file pone.0356261.s004.zip › Data and Code/RF/Pinus yunnanensis forest/figures/PDP_N.P_AK.pdf]

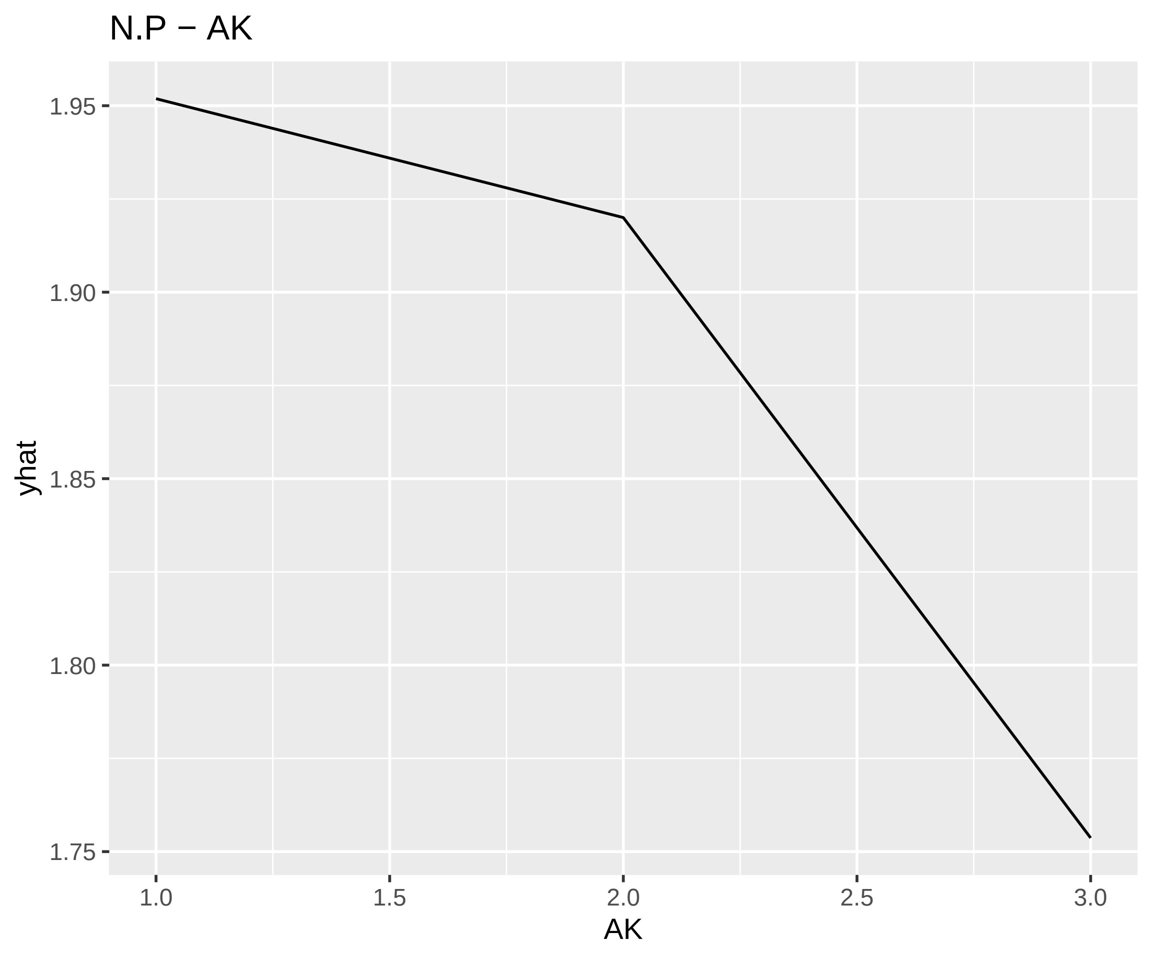
N.P – AK
yhat
AK
1.95
1.90
1.85
1.80
1.75
1.0
1.5
2.0
2.5
3.0
